# Self-efficacy to reduce sedentary behavior status and its influencing factors among middle-aged or older adults with chronic diseases: a cross-sectional study

**DOI:** 10.1186/s12889-025-25094-w

**Published:** 2025-12-01

**Authors:** Yuxi Chen, Jufeng Chen, Sunanjie Zhao, Peiya Tao, Guohu Han, Zhuo Wang

**Affiliations:** 1 School of Medical and Health Engineering, Changzhou University, Changzhou, Jiangsu China; 2 Department of Nursing, Changzhou Second People’s Hospital, Affiliated Hospital of Nanjing Medical University, Changzhou, China

**Keywords:** Sedentary behavior, Self-efficacy, Middle-aged and elderly, Chronic diseases, Influencing factors

## Abstract

**Background:**

Sedentary behavior (SB) is an important public health risk factor and is common among people with chronic diseases. Self-efficacy to reduce SB represents a distinct construct. Nonetheless, studies investigating interventions for SB are lacking, which may be addressed in future research. Hence, understanding the factors influencing self-efficacy to reduce SB among middle-aged and elderly people with chronic diseases holds significance.

**Methods:**

This study included 583 middle-aged and elderly people from March 2024-May 2024 in 6 communities in Changzhou. The Chinese version of the self-efficacy to reduce sedentary behavior (SRSB) questionnaire was used to measure individuals’ confidence in reducing SB. Data were analyzed using SPSS 27.0 using descriptive statistics, cluster analysis, chi-square test, and ordered multinomial logistic regression to explore the influencing factors of SRSB in people with chronic diseases.

**Results:**

A survey completion rate of 97.2% was achieved. A total of 583 people from 6 communities participated in this study. The median (interquartile range, IQR) score of the Chinese version of the SRSB was 3.78 (3.00–4.44), with a range of 1.0 to 5.0. Factors associated with the self-efficacy to reduce SB were body mass index (BMI), education level, number of chronic diseases, smoking status, and physical exercise.

**Conclusions:**

The findings of this study confirmed that the self-efficacy to reduce SB of people with chronic diseases in China was at a moderate level. In health management, emphasis should be placed on overweight or obese individuals, poorly educated, having more chronic diseases, smoking, and not exercising. Health education should focus on preventive interventions against SB, promoting the adoption of beneficial lifestyles for middle-aged and elderly people with chronic diseases.

**Supplementary Information:**

The online version contains supplementary material available at 10.1186/s12889-025-25094-w.

## Introduction

With the aging population, the prevalence of chronic diseases and comorbidity patterns pose significant challenges to global health, and the burden of chronic diseases shows a dramatically increasing trend worldwide. According to the World Health Organization, 74% of global deaths are attributed to chronic diseases [1]. Furthermore, chronic diseases are the primary cause of early death among adults worldwide, with older adults being more prone to developing most chronic diseases compared to younger adults [2]. According to the World Health Organization’s global report [1], 80% of chronic disease deaths occur in low- and middle-income countries. Evidence revealed that chronic diseases were highly prevalent among older adults in China and varied geographically [3]. According to the latest 2020 data released by the China Health and Retirement Longitudinal Study (CHARLS), common chronic diseases among middle-aged and elderly people in China include hypertension, diabetes, arthritis or rheumatism, stomach or digestive diseases, dyslipidemia, heart disease, and other conditions. The prevalence of chronic diseases in this population has reached 80.9%, with a comorbidity rate of 58.1% [4]. Zhang et al. [5] conducted a survey of 10,197 middle-aged and older adults and found that the prevalence of multimorbidity in this population was 55.12%, and 65.60% among people aged ≥ 65 years. Chronic diseases seriously threaten the health of Chinese residents and constitute a major public health problem affecting the social and economic development of China.

Sedentary behavior (SB) is an independent risk factor for chronic diseases and has emerged as the focus of research, clinical, and policy interest [6]. The Physical Activity and Sedentary Behavior Guidelines issued by the WHO in 2020 clearly state that people with chronic diseases should limit SB [7]. SB can be defined as any behavior in which the energy expenditure is less than 1.5 metabolic equivalents (METs) while sitting or lying down in an awake activity [8]. SB is common in patients with chronic diseases and affects the care and prognosis of the disease. Several studies have reported that patients with type 2 diabetes spend at least 50% of their total lifetime in sedentary behavior [9]. Moreover, COPD patients with an average age of 65 years are sedentary for at least 10 h per day [10], and the average daily SB time of stroke patients is (479.65 ± 112.65) min [11]. A large number of studies have confirmed that SB is associated with adverse health outcomes such as cardiovascular disease, all-cause mortality, type 2 diabetes, and cancer [12–14]; in addition, sitting for an extended period can also increase the risk of chronic disease and death, seriously affecting the quality of life of the patients with chronic diseases [15]. Previous research has reported that even physical activity at the recommended level does not offset the harm of sedentary behavior [16]. Additionally, SB tends to be unintentional and often occurs in conjunction with other behaviors, such as reading books, watching TV, and socializing [17]. Similar to smoking, SB has a delayed impact, and people may be exposed to potential health risks later in their lives. Therefore, reducing the SB of patients with chronic diseases is crucial.

Self-efficacy has been shown to be a reliable predictor of diverse health behaviors [18], and is usually conceptualized as situation- or domain-specific. Self-efficacy is a psychological concept defined as an individual’s belief in his or her capacity to execute behaviors necessary to produce specific performance attainments [19]. A substantial body of evidence has repeatedly demonstrated a strong link between self-efficacy and physical activity (PA) levels [20–22]. PA and self-efficacy are positively correlated [23], and exercise self-efficacy can predict subsequent PA [24]. A systematic review indicated that SB is significantly negatively associated with PA, particularly because SB often displaces time that could otherwise be used for light intensity activity, thereby reducing individuals’ overall PA levels [25]. However, the research on the relationship between self-efficacy to reduce SB and SB is still in the preliminary exploration stage. SB and PA, as two different behavioral patterns, should be explained by different predictors. Whipple stated that self-efficacy to reduce SB represents a distinct construct [26] and could be part of future research into interventions for SB. Therefore, exploring SB-specific self-efficacy is indispensable for reducing SB.

Based on Social Cognitive Theory, self-efficacy is regarded as one of the most central cognitive determinants of behavior change, and the influence of sociostructural factors on behavior is primarily exerted through their impact on self-efficacy beliefs [27]. Moreover, illness and treatment can also affect patients’ levels of exercise self-efficacy [28]. Therefore, we hypothesize that self-efficacy to reduce SB may also be affected by sociodemographic characteristics and chronic disease status. At present, studies exploring the related factors impacting the self-efficacy to reduce SB are scarce. Given the critical role of self-efficacy to reduce SB in predicting SB, identifying its influencing factors is of great significance for developing effective interventions to reduce sedentary time among patients with chronic diseases. Consequently, the present study aimed to assess the status of self-efficacy to reduce SB in people with chronic diseases in China and to investigate its influencing factors, in order to inform the design of future tailored intervention strategies for patients with chronic diseases.

## Methods

### Study participants and design

This study adopted a cross-sectional design. The project was carried out in 6 communities in Changzhou, PR China, from March to May 2024. The inclusion criteria were the following: (1) age ≥ 45 years old; (2) have clear consciousness, good language communication ability; (3) willing to participate in this study. The exclusion criteria were as follows: (1) mental illness or cognitive dysfunction; (2) suffering from other diseases resulting in limited PA or the need for wheelchair assistance out; (3) inability to communicate due to serious diseases, such as heart failure and respiratory failure. Based on the literature review, 12 independent variables were initially developed. The sample size was increased by 20% to account for a potential rejection rate and an invalid questionnaire rate of 20%, resulting in the required sample size of 150 ~ 225. In the sampling process, to ensure that the sample size meets the minimum standards, a larger number of middle-aged and elderly people with chronic diseases were included in the survey.

### Measures

#### Demographic characteristics

Demographic characteristics included age, gender, BMI, place of residence, education level, living conditions, marital status, monthly income, working status, number of chronic diseases, smoking status, and lack of physical activity. In this study, individuals engaging in moderate or higher intensity PA at least 3 times per week for no less than 30 min per session or those performing moderate to heavy manual labor are considered physically active [29]. Specifically, moderate physical labor includes activities such as regular lifting, weeding, painting, and picking fruits and vegetables. Heavy labor includes more intense tasks like lifting heavy objects, shoveling, forging, or digging.

#### Self-efficacy to reduce sedentary behavior (SRSB)

The SRSB questionnaire was used to assess the confidence of chronic disease patients to reduce SB. Originally developed by Whipple et al. [26] in 2023, the SRSB demonstrated strong psychometric properties in adult samples aged 18 to 75 years, with a Cronbach α of 0.920. Confirmatory factor analysis supported a unidimensional model. The scale comprises nine items scored on a 5-point Likert scale ranging from ‘strongly disagree’ (1 point) to ‘strongly agree’ (5 points). A total score ranging between 9 and 45 points was obtained, with higher scores demonstrating higher confidence in reducing SB. In this study, we utilized the Chinese version of the SRSB, which was validated by our research team in previous studies [30]. This scale was translated using the Brislin back-translation method and further refined through expert consultation (see supplementary materials). The Chinese version of the scale showed a Cronbach α of 0.921 and a retest reliability of 0.951. In this study, the Cronbach α for the Chinese version of the SRSB was 0.920, which supported the reliability of this scale in middle-aged and elderly patients with chronic diseases.

### Data collection

Data collection was completed by three experienced researchers, and each participant gave their informed consent. The objective, significance, and methods of this study were explained to the participants at the outset. Before the completion of the questionnaires, all participants were fully informed about the purpose of this study, the right to refuse participation in the study, and the method of anonymous data collection. Participants were required to fill out questionnaires, but if the participant was unable to read or write, the researcher would administer the survey in person while assisting the patient to comprehend the questions. During the filling process, the researchers avoided any inductive explanation. Clarification was provided by the investigator if participants reported difficulties in understanding any statements. After the participants answered, the researchers assisted the participants in filling in the facts and checked them immediately to guarantee the accuracy and completeness of the data.

### Statistical analysis

SPSS software (version 27.0) was used for statistical analysis. The data normality was evaluated using the Kolmogorov–Smirnov test (*P* > 0.05 for all variables) and Q-Q normality plots. The data of all variables did not conform to the normal distribution and were analyzed using non-parametric methods. Continuous variables are presented as median (IQR), and categorical variables are shown as numbers (%). The scores of the SRSB were classified by cluster analysis. First, the between-group linkage method in hierarchical clustering was used to generate a dendrogram and determine the possible number of clusters. Then, the optimal number of clusters was determined using the iterative partitioning method of K-means clustering. Each cluster was required to contain more than 5% of the total sample size. The chi-square test was used to compare differences in SRSB among groups. In addition, ordered logistic regression was conducted to analyze the factors influencing SRSB in middle-aged and elderly people. For all analyses, a *p*-value < 0.05 was considered statistically significant (two-tailed).

## Results

### Descriptive characteristics of participants

A total of 600 questionnaires were distributed in this study, and 583 valid questionnaires were recovered, with an effective recovery rate of 97.2%. Most of the people with chronic diseases were female (53.7%) and over 60 years old (54.4%). Table 1 provides facts about middle-aged and elderly people with chronic diseases.

**Table 1:** Univariate analysis of the influencing factors of the SRSB in people with chronic diseases

Variable	*N* (%)	Low-level group(*n* = 64)	Medium-level group(*n* = 240)	High-level group(*n* = 279)	χ²	*P*
Age (years)					3.621	0.164
45ཞ59	266 (45.6%)	23	107	136		
≥ 60	317 (54.4%)	41	133	143		
Gender					4.685	0.096
Male	270 (46.3%)	37	113	120		
Female	313 (53.7%)	27	127	159		
BMI (kg/m^2^)					14.503	0.006**
< 18.5	14 (2.4%)	5	8	1		
18.5–23.9	289 (49.6%)	29	114	146		
> 24.0	280 (48.0%)	30	118	132		
Place of residence					8.361	0.015*
City	471 (80.8%)	50	182	239		
Rural	112 (19.2%)	14	58	40		
Education level					13.617	0.034*
Primary school or below	120 (20.6%)	19	51	50		
Junior high school	250 (42.9%)	25	110	115		
High school	122 (20.9%)	11	54	57		
College or above	91 (15.6%)	9	25	57		
Work status					14.658	< 0.001**
Full time	207 (35.5%)	9	89	109		
Unemployed	376 (64.5%)	55	151	170		
Marital status					2.630	0.268
Married (Spouse in tow)	516 (88.5%)	53	212	251		
Single(divorced/widowed/unmarried)	67 (11.5%)	11	28	28		
Monthly income (yuan)					10.914	0.028*
< 3k	239 (41.0%)	32	108	99		
3-6k	197 (33.8%)	13	76	108		
> 6k	147 (25.2%)	19	56	72		
Residence status					12.73	0.044*
Living alone	53 (9.09%)	8	22	23		
Living with children	86 (14.75%)	7	27	52		
Living with a spouse	334 (57.29%)	41	140	153		
Living with children and spouse	110 (18.87%)	8	51	51		
Number of chronic diseases					20.090	< 0.001**
Without chronic diseases	236 (40.5%)	14	89	133		
1 ~ 2 chronic diseases	292 (50.1%)	38	128	126		
≥ 3 chronic diseases	55 (9.4%)	12	23	20		
Whether or smoke					11.930	0.003**
Yes	143 (24.5%)	25	64	54		
No	440 (75.5%)	39	176	225		
Whether lack of physical activity					56.254	< 0.001**
Yes	225 (38.6%)	48	104	73		
No	358 (61.4%)	16	136	206		

due to rounding to one decimal place, the total percentage may not add up exactly to 100%; therefore, the percentages for some categories are displayed to two decimal places

* *P* < 0.05

** *P* < 0.01

### Cluster analysis result

According to the cluster analysis dendrogram, 583 samples can be divided into 2, 3, and 4 categories, all meeting the requirement of the number of samples in each cluster exceeding 5% of the total number of samples. Considering the interpretability and practical significance of clustering and the suggestions of experts in related fields, the optimal k value was determined to be 3. Then, the K-means clustering method was utilized to set the maximum number of clusters as 10, and the SRSB scores were set as the clustering variable. According to the characteristics of each category, group 1 was named the ‘low-level group’, with 64 cases (11.0%); group 2 was the ‘medium-level group’, with 240 cases (41.2%); group 3 was the ‘high-level group’, with 279 cases (47.9%).

### Univariate analysis of influencing factors of the SRSB in patients with chronic diseases

In the comparison of the SRSB among middle-aged and elderly patients with chronic diseases, factors such as BMI, residence place, education level, work status, monthly income, residence status, number of chronic diseases, smoking status, and lack of physical exercise showed statistically significant differences (*P* < 0.05). In contrast, age, gender, and marital status showed no statistically significant difference (Table 1).

### The results of ordered multinomial logistic regression analysis of the influencing factors of the SRSB in patients with chronic diseases

The clustering results of the SRSB in people with chronic diseases were set as the dependent variable (low-level group = 1, medium-level group = 2, high-level group = 3). Subsequently, the independent variables with statistically significant differences in Table 1 were used for ordered multi-classification logistic regression analysis, as shown in Table 2. The likelihood-ratio test revealed that χ^2^ = 101.349, *P* < 0.001; the goodness of fit test showed that χ^2^ = 789.446, *P =* 0.959; the test of parallel lines showed that χ^2^ = 24.738, *P =* 0.075. These results showed that the ordered multi-classification logistic regression analysis was feasible and the model was meaningful. The results of the ordinal logistic regression analysis indicated that BMI, education level, number of chronic diseases, smoking, and lack of physical exercise influenced the score of the SRSB in patients with chronic diseases (*P* < 0.05). Compared with overweight or obese patients with a college degree or above, non-smoking, and physical exercise, the scores of the SRSB were lower in those who were underweight, with primary school and below and junior high school, smoking, and lack of physical exercise (*P* < 0.05); compared with those with more than three kinds of chronic diseases, those without any diseases demonstrate a higher score (*P* < 0.05), as shown in Table 3.

**Table 2:** Variable assignment

Variable	Assignment
BMI	< 18.5 = 1, 18.5–23.9 = 2, > 24.0 = 3
Place of residence	Rural (0, 0), City (0, 1)
Education level	Primary school and below = 1, Junior high school = 2, High school = 3, College or above = 4
Work status	Unemployed (0, 0), Full time (0, 1)
Monthly income	< 3k = 1, 3-6k = 2, >6k = 3
Residence status	Living alone (0, 0, 0, 0), Living with children (0, 1, 0, 0), Living with a spouse (0, 0, 1, 0), Living with children and spouse (0, 0, 0, 1)
Number of chronic diseases	Without chronic diseases = 1, 1 ~ 2 chronic diseases = 2, ≥ 3 chronic diseases = 3
Whether or smoke	Yes = 0, No = 1
Whether lack of physical activity	Yes = 0, No = 1

**Table 3:** Multivariate ordinal logistic regression analysis of the influencing factors of the SRSB among people with chronic diseases

Variable	Eigenvalue	Reference group	β	SE	Wald χ^2^	*P*	OR (95%CI)
BMI (kg/m^2^)	< 18.5	> 24.0	−1.472	0.555	7.026	0.008*	0.229(0.077, 0.681)
	18.5–23.9		0.058	0.172	0.114	0.736	1.060(0.757, 1.486)
Place of residence	City	Rural	0.163	0.217	0.563	0.453	1.177(0.770, 1.799)
Education level	Primary school and below	College or above	−0.679	0.327	4.318	0.038*	0.507(0.267, 0.962)
	Junior high school		−0.581	0.285	4.155	0.042*	0.559(0.320, 0.978)
	High school		−0.34	0.296	1.32	0.251	0.712(0.399, 1.271)
Work status	Full time	Unemployed	0.326	0.192	2.891	0.089	1.385(0.951, 2.020)
Monthly income	< 3k	> 6k	−0.067	0.244	0.075	0.785	0.935(0.580, 1.508)
	3-6k		0.321	0.235	1.859	0.173	1.379(0.869, 2.186)
Residence status	Living alone	Living with children and spouse	−0.014	0.344	0.002	0.968	0.986(0.502, 1.937)
	Living with children		0.464	0.307	2.291	0.13	1.590(0.872, 2.904)
	Living with a spouse		−0.068	0.226	0.091	0.763	0.934(0.600, 1.455)
Number of chronic diseases	Without chronic diseases	≥ 3 chronic diseases	0.624	0.306	4.157	0.041*	1.866(1.024, 3.401)
	1 ~ 2 chronic diseases		0.237	0.289	0.671	0.413	1.267(0.719, 2.232)
Whether or smoke	Yes	No	−0.421	0.197	4.588	0.032*	0.656(0.446, 0.965)
Whether lack of physical activity	Yes	No	−1.099	0.176	38.833	< 0.001**	0.333(0.236, 0.471)

BMI(kg/m^2^)* classification standard*, <18.5 *underweight*, 18.5ཞ23.9 *normal weight*, 24.0ཞ27.9 *overweight*, ≥ 28.0 *obesity,* smoke: continuous or cumulative smoking for 6 months or more; physical activity: at least 30 min of physical exercise, more than 3 times per week; regular lifting, weeding, painting, picking fruits and vegetables, etc., are considered not lack of physical activity

* *P* < 0.05

** *P* < 0.01

## Discussion

The trend of chronic diseases and comorbidities in middle-aged individuals and the elderly has emerged as a public health problem in China and throughout the world. Furthermore, SB is closely associated with the incidence and mortality rates of various chronic diseases [31]. Given the detrimental impact of sedentary behavior on the health of individuals with chronic diseases, it is particularly important to explore effective intervention strategies. Self-efficacy has long been regarded as one of the key variables influencing behavior change [32]. As an emerging construct, SRSB shows potential as a critical factor for promoting behavior change. Although previous studies have suggested that SRSB could be incorporated into future SB interventions [26], empirical research is scarce. Therefore, the importance of timely assessment of the SRSB among chronic patients cannot be over-emphasized. To date, this was the first study to investigate the level of SRSB among people with chronic disease in China. A key finding from this study showed that the predictors of chronic patients’ self-efficacy to reduce SB included BMI, education level, number of chronic diseases, smoking status, and physical exercise. These findings provide a reference for developing individualized strategies to help people with chronic diseases reduce SB.

In our study, across the entire sample (*n* = 583), the M (IQR) was 3.78 (3.00–4.44), with a range of 1.0 to 5.0. Compared with the median of the total score of the scale, the results of our study were in the middle level. These results are consistent with a previous study [26]. Currently, the development of the SRSB has not been standardized, and the item-level scores in our sample were largely consistent. Hence, the results were grouped artificially through cluster analysis. In the low-level group, item 4 scored the lowest, and item 5 scored the highest, while in the middle-level group and the high-level group, item 1 scored the highest and item 9 scored the lowest. The results of the low-level group may be attributed to a larger proportion of patients with more chronic diseases in the low-level group, leading to more time spent resting. Moreover, the environment at home is more comfortable, and reducing SB at home requires more self-discipline. However, such individuals may have more opportunities to engage in various activities while going out, so changes in the environment may promote activities to reduce SB [33], such as parks and walking trails. The results of the medium-level and high-level groups are related to stronger health awareness and behavioral control capabilities, as these individuals have more opportunities and conditions to choose to stand in their work or life. However, environmental restrictions and transport (such as cars and airplanes) also limit the possibility of standing.

This study revealed that underweight people reported lower levels of SRSB, which is consistent with previous research [26]. Overweight or obese patients may be aware of their high health risks, such as cardiovascular disease and diabetes, and have a stronger incentive to take measures to reduce SB to improve their health. In contrast, underweight people may think that their health risks are low and lack the motivation to reduce SB. Additionally, overweight or obese people may be subjected to more pressure and social support from society, family, and friends, prompting them to address their SB more actively. Conversely, underweight people may not be exposed to the same degree of social support and pressure to change their behavior, especially when they have no obvious health problems. Research has also shown that [34] weight does not affect self-efficacy, and overweight or obese people are less likely to seek weight loss help. In other words, this group of people may not pay much attention to their weight changes because of their high general self-efficacy. Therefore, despite being overweight or obese for extended periods, their scores on the SRSB remain high.

This study also reveals that education level was associated with levels of the SRSB, with higher education level being associated with a higher SRSB score. The education level can be used as an indicator to measure the individuals’ socio-economic status [35]. Individuals with higher socio-economic status have higher autonomy in choosing different lifestyles, often have higher health cognitive ability, and have a strong subjective willingness to choose a good lifestyle [36]. Compared with people with a primary school education and below, people with a college or university education and above generally attain more health knowledge and awareness. Therefore, these people may be more aware of the negative impact of SB on health and are more aware of the importance of reducing SB. Furthermore, research has shown that education level is the influencing factor of health behavior in hypertensive elderly patients [37]; patients with higher education levels exhibit better health behaviors in terms of reducing SB. In addition, people with higher education levels may live in a healthier environment, which encourages an active lifestyle. For example, some workplaces provide standing desks to reduce SB [38–40], which may lead to higher self-efficacy. Generally, patients with a higher education level have a stronger ability to absorb and master knowledge. Such individuals can efficiently use information resources to obtain accurate and scientific medical knowledge, developing a deeper understanding of their health status. This cognitive advantage enables them to face the challenges associated with their disease, allowing a more positive view of health behaviors such as reducing SB.

Findings from this study also indicate that the SRSB score of people with ≥ 3 chronic diseases was lower than that of people without chronic diseases. Previous studies have reported a higher incidence of SB in patients with chronic diseases [10, 41–43], which often coexist and interact with each other. Research has shown that the incidence of various chronic diseases increases as age increases, including cardiovascular disease, diabetes, osteoarthritis, and cancer [44–47]. As the number of diseases, sedentary time also tends to increase [48]. In addition, the coexistence of multiple diseases may lead to anxiety and depression. Research has shown that [49] the number of diseases is an influencing factor for depression; as the number of chronic diseases increases, the possibility of depression also increases. Individuals suffering from ≥ 5 chronic diseases have a 4 times higher risk of depression compared to people who are not sick. Depression patients may lack motivation for PA, thereby affecting self-efficacy and increasing SB. Therefore, the SRSB of people with a large number of diseases is lower than that of people without chronic diseases. A systematic review of qualitative research revealed [50] some misunderstandings in the elderly, such as ‘those with complications should be sedentary’ and ‘physical weakness caused by age growth cannot be avoided’, which hinders the adoption of SB-reducing activities in the elderly. This may partially be attributed to muscle atrophy in the elderly. Such changes limit the activities of elderly patients with chronic diseases, resulting in an increase in SB. Hence, for the population with more diseases, a lower SRSB score is associated with a higher difficulty to reduce SB, raising the difficulty in forming and maintaining good behavior habits.

The present study revealed that the SRSB score of non-smokers was higher than that of smokers. Non-smokers may have stronger health awareness and self-management ability. Research has shown that [51] non-smokers have a healthier overall lifestyle. As the score of healthy lifestyle increases, the proportion of smokers gradually decreases, with non-smokers in the high score group of healthy lifestyle. According to Yuan et al. [52], a history of smoking is negatively correlated with the level of self-efficacy in elderly patients with chronic diseases, which may be related to poor awareness of health management and a lack of self-care beliefs. Although elderly patients are aware of the dangers of smoking, their confidence in self-health management is reduced due to withdrawal symptoms and psychological cravings. Moreover, non-smokers may have developed a healthy lifestyle, which includes less sedentary time and more PA, which prompts them to adopt healthy behaviors in other aspects, such as reducing SB. Research has shown that [53] smokers often have poor health behaviors. A 7-year prospective observational research indicated that [54] smoking is associated with a lack of exercise, and smokers often lack exercise, which may be more likely to lead to sedentary behavior. Furthermore, the SRSB score of people who lack physical exercise was lower than that of people who do not lack physical exercise. People who exercise regularly are speculated to have stronger health awareness, are more concerned about their health, and are more likely to participate in various activities to reduce SB. People who lack physical exercise are often accustomed to a sedentary lifestyle, which makes it difficult for such people to confidently believe that they can reduce SB.

### Limitations

Nevertheless, several limitations of the present study should be acknowledged. Firstly, this study adopted a cross-sectional design, which limits causal inference. Secondly, due to practical constraints in the study environment, objective tools were not employed to assess PA and SB, and comprehensive, accurate information on participants’ chronic disease status could not be fully obtained. Moreover, environmental factors such as digital media use, residential settings, and transportation that may influence SRSB were not assessed and should be considered in future research. Therefore, further research is needed to identify additional influencing factors. Thirdly, this study included only patients with chronic diseases in a specific city, which may not be fully representative of the broader or geographically diverse population of patients with chronic diseases. Thus, multi-center, multi-regional studies should be conducted to generalize the findings in future studies. Finally, due to the nature of self-reported measures, participants may have inaccurately reported their abilities.

## Conclusion

In conclusion, this study demonstrated the self-efficacy to reduce SB of people with chronic diseases in China was at a moderate level and related to many factors. In daily life, the adverse effects of SB accumulate over time, and even when people are aware of the harms, it can be difficult to make changes due to a lack of effective methods, poor time management, or environmental constraints. The importance of modifiable factors in health promotion should be emphasized. In health management, emphasis should be placed on people who are overweight or obese, poorly educated, have more chronic diseases, smoke, and do not exercise, thereby strengthening health education and prevention interventions on sedentary behavior. At present, few studies have investigated self-efficacy to reduce SB. Therefore, the next study will use a wider sample and more comprehensive data collection methods to deeply explore the factors affecting self-efficacy to reduce SB and provide a reference for future interventions.

## Supplementary Information

Supplementary Material 1.

## Data Availability

The datasets used and analyzed in the current study are available from the corresponding author upon reasonable request.

## Abbreviations

IQR: Interquartile Range
CHARLS: China Health and Retirement Longitudinal Study
SB: Sedentary Behavior
PA: Physical Activity
WHO: World Health Organization
METs: Metabolic Equivalents
SRSB: Self-efficacy to Reduce Sedentary Behavior

## References

[1] World Health Organization. Noncommunicable diseases. Published September 18. 2022. Accessed November 30, 2024. https://www.who.int/news-room/fact-sheets/detail/noncommunicable-diseases

[2] Bauer UE, Briss PA, Goodman RA, Bowman BA. Prevention of chronic disease in the 21st century: elimination of the leading preventable causes of premature death and disability in the USA. Lancet. 2014;384(9937):45–52. 10.1016/S0140-6736(14)60648-6. 24996589 10.1016/S0140-6736(14)60648-6

[3] Su B, Li D, Xie J, et al. Chronic disease in china: geographic and socioeconomic determinants among persons aged 60 and older. J Am Med Dir Assoc. 2023;24(2):206-e2125. 10.1016/j.jamda.2022.10.002. 36370750 10.1016/j.jamda.2022.10.002

[4] Zhang H, Zhao G, Liu A, et al. Analysis of chronic comorbidities patterns and gender differences among middle-aged and elderly people in China. Zhengzhou Daxue Xuebao (Yixue Ban). 2025;60(2):186–9. 10.13705/j.issn.1671-6825.2024.04.056. Chinese.

[5] Zhang Y, Zhou L, Liu S, et al. Prevalence, correlates and outcomes of multimorbidity among the middle-aged and elderly: findings from the China health and retirement longitudinal study. Arch Gerontol Geriatr. 2020;90. 10.1016/j.archger.2020.104135. 32554217 10.1016/j.archger.2020.104135

[6] Rawlings GH, Williams RK, Clarke DJ, et al. Exploring adults’ experiences of sedentary behaviour and participation in non-workplace interventions designed to reduce sedentary behaviour: a thematic synthesis of qualitative studies. BMC Public Health. 2019;19. 10.1186/s12889-019-7365-1. 31409324 10.1186/s12889-019-7365-1 PMC6692932

[7] Bull FC, Al-Ansari SS, Biddle S, et al. World health organization 2020 guidelines on physical activity and sedentary behaviour. Br J Sports Med. 2020;54(24):1451–62. 10.1136/bjsports-2020-102955. 33239350 10.1136/bjsports-2020-102955 PMC7719906

[8] Tremblay MS, Aubert S, Barnes JD, SBRN Terminology Consensus Project Participants, et al. Sedentary behavior research network (SBRN) – terminology consensus project process and outcome. Int J Behav Nutr Phys Act. 2017;14(1):75. 10.1186/s12966-017-0525-8. 28599680 10.1186/s12966-017-0525-8 PMC5466781

[9] De Greef KP, Deforche BI, Ruige JB, et al. The effects of a pedometer-based behavioral modification program with telephone support on physical activity and sedentary behavior in type 2 diabetes patients. Patient Educ Couns. 2011;84(2):275–9. 10.1016/j.pec.2010.07.010. 20732776 10.1016/j.pec.2010.07.010

[10] Orme MW, Steiner MC, Morgan MD, et al. 24-hour accelerometry in COPD: exploring physical activity, sedentary behavior, sleep and clinical characteristics. COPD. 2019;14:419–30. 10.2147/COPD.S183029. 10.2147/COPD.S183029 PMC6388788 30863042

[11] Wei J, Yang F, Dong X. Perceived social support on objective measured sedentary behavior of stroke patients: the mediating role of exercise self-efficacy. Front Psychol. 2024;15. 10.3389/fpsyg.2024.1444214. 39386140 10.3389/fpsyg.2024.1444214 PMC11461327

[12] Young DR, Hivert MF, Alhassan S, et al. Sedentary behavior and cardiovascular morbidity and mortality: A science advisory from the American heart association. Circulation. 2016;134(13). 10.1161/CIR.0000000000000440. 10.1161/CIR.0000000000000440 27528691

[13] de Rezende LFM, Rodrigues Lopes M, Rey-López JP, Matsudo VKR, Luiz OdoC. Sedentary behavior and health outcomes: an overview of systematic reviews. PLoS ONE. 2014;9(8). 10.1371/journal.pone.0105620. 25144686 10.1371/journal.pone.0105620 PMC4140795

[14] Gilchrist SC, Howard VJ, Akinyemiju T, et al. Association of sedentary behavior with cancer mortality in Middle-aged and older US adults. JAMA Oncol. 2020;6(8):1. 10.1001/jamaoncol.2020.2045. 10.1001/jamaoncol.2020.2045 PMC7303924 32556069

[15] Kim Y, Lee E. The association between elderly people’s sedentary behaviors and their health-related quality of life: focusing on comparing the young-old and the old-old. Health Qual Life Outcomes. 2019;17(1). 10.1186/s12955-019-1191-0. 31349858 10.1186/s12955-019-1191-0 PMC6660966

[16] Rollo S, Gaston A, Prapavessis H. Cognitive and motivational factors associated with sedentary behavior: a systematic review. AIMS Public Health. 2016;3(4):956–84. 10.3934/publichealth.2016.4.956. 29546206 10.3934/publichealth.2016.4.956 PMC5690416

[17] Gardner B, Flint S, Rebar AL, et al. Is sitting invisible? Exploring how people mentally represent sitting. Int J Behav Nutr Phys Act. 2019;16(1). 10.1186/s12966-019-0851-0. 31606040 10.1186/s12966-019-0851-0 PMC6790031

[18] Sheeran P, Montanaro E, Bryan A, et al. The impact of changing attitudes, norms, and self-efficacy on health-related intentions and behavior: a meta-analysis. Health Psychol. 2016;35(11):1178–88. 10.1037/hea0000387. 27280365 10.1037/hea0000387

[19] Bandura A. Self-efficacy: toward a unifying theory of behavioral change. Psychol Rev. 1977;84(2):191–215. 847061 10.1037//0033-295x.84.2.191

[20] Alrimali AM. Assessment of physical activity level, self-efficacy and perceived barriers to physical activity among adult Saudi women. J Taibah Univ Med Sci. 2023;18(4):812–21. 10.1016/j.jtumed.2022.12.017. 36852249 10.1016/j.jtumed.2022.12.017 PMC9957769

[21] Kaewthummanukul T, Brown KC. Determinants of employee participation in physical activity: critical review of the literature. AAOHN J. 2006;54(6):249–61. 10.1177/216507990605400602. 16800402 10.1177/216507990605400602

[22] McAuley E, Blissmer B, Katula J, Duncan TE, Mihalko SL. Physical activity, self-esteem, and self-efficacy relationships in older adults: A randomized controlled trial. Ann Behav Med. 2000;22(2):131–9. 10.1007/BF02895777. 10962706 10.1007/BF02895777

[23] Pan SY, Cameron C, DesMeules M, Morrison H, Craig CL, Jiang X. Individual, social, environmental, and physical environmental correlates with physical activity among canadians: a cross-sectional study. BMC Public Health. 2009;9:21. 10.1186/1471-2458-9-21. 19149865 10.1186/1471-2458-9-21 PMC2639577

[24] Cabral DF, Fried PJ, Bigliassi M, Cahalin LP, Gomes-Osman J. Determinants of exercise adherence in sedentary middle‐aged and older adults. Psychophysiology. 2024;61(9):e14591. 10.1111/psyp.14591. 38629783 10.1111/psyp.14591 PMC11330369

[25] Mansoubi M, Pearson N, Biddle SJH, Clemes S. The relationship between sedentary behaviour and physical activity in adults: A systematic review. Prev Med. 2014;69:28–35. 10.1016/j.ypmed.2014.08.028. 25193005 10.1016/j.ypmed.2014.08.028

[26] Whipple MO, Bergouignan A, Hooker SA. Development and initial validation of a measure to assess Self-Efficacy to reduce sedentary behavior. Med Sci Sports Exerc. 2023;55(10):1933–9. 10.1249/MSS.0000000000003215. 37227220 10.1249/MSS.0000000000003215 PMC10524260

[27] Bandura A. Health promotion by social cognitive means. Health Educ Behav. 2004;31(2):143–64. 10.1177/1090198104263660. 15090118 10.1177/1090198104263660

[28] Li X, Geng L, Yuan Q, Yue S. Relationship between self-efficacy and physical activity among colorectal cancer patients: A cross-sectional study. Nurs Open. 2023;10(6):3613–21. 10.1002/nop2.1608. 36611230 10.1002/nop2.1608 PMC10170901

[29] Wang X, Li W, Song F, et al. Carotid atherosclerosis detected by ultrasonography: A National Cross-Sectional study. J Am Heart Assoc. 2018;7(8):e008701. 10.1161/JAHA.118.008701. 29622590 10.1161/JAHA.118.008701 PMC6015437

[30] Wang Z, Chen YX, Zhu SX, et al. Chinesization of the Self-Efficacy to reduce sedentary behavior scale and its reliability and validity test. Chin Nurs Res. 2025;39(10):1669–74. 10.12102/j.issn.1009-6493.2025.10.012. [in Chinese].

[31] Wilmot EG, Edwardson CL, Achana FA, et al. Sedentary time in adults and the association with diabetes, cardiovascular disease and death: systematic review and meta-analysis. Diabetologia. 2012;55(11):2895–905. 10.1007/s00125-012-2677-z. 22890825 10.1007/s00125-012-2677-z

[32] Szczuka Z, Banik A, Abraham C, Kulis E, Luszczynska A. Associations between self-efficacy and sedentary behaviour: a meta-analysis. Psychol Health. 2021;36(3):271–89. 10.1080/08870446.2020.1784419. 32597242 10.1080/08870446.2020.1784419

[33] Kaushal N, Rhodes RE. The home physical environment and its relationship with physical activity and sedentary behavior: A systematic review. Prev Med. 2014;67:221–37. 10.1016/j.ypmed.2014.07.026. 25084562 10.1016/j.ypmed.2014.07.026

[34] Schmidt G, Pichler S. General Self-Efficacy and body weight: the role of race and gender. Psychol Rep. 2021;124(6):2476–500. 10.1177/0033294120961072. 32998657 10.1177/0033294120961072

[35] Zhang YB, Chen C, Pan XF, et al. Associations of healthy lifestyle and socioeconomic status with mortality and incident cardiovascular disease: two prospective cohort studies. BMJ. 2021;373:n604. 10.1136/bmj.n604. 33853828 10.1136/bmj.n604 PMC8044922

[36] Dahl E. Social mobility and health: cause or effect? BMJ: Br Med J. 1996;313(7055):435. 10.1136/bmj.313.7055.435. 8776298 10.1136/bmj.313.7055.435 PMC2351864

[37] Li Sxia, Zhang L. Health behavior of hypertensive elderly patients and influencing factors. Aging Clin Exp Res. 2013;25(3):275–81. 10.1007/s40520-013-0051-8. 23740592 10.1007/s40520-013-0051-8

[38] Neuhaus M, Eakin EG, Straker L, et al. Reducing occupational sedentary time: a systematic review and meta-analysis of evidence on activity‐permissive workstations. Obes Rev. 2014;15(10):822–38. 10.1111/obr.12201. 25040784 10.1111/obr.12201

[39] Gilson ND, Suppini A, Ryde GC, Brown HE, Brown WJ. Does the use of standing hot desks change sedentary work time in an open plan office? Prev Med. 2012;54(1):65–7. 10.1016/j.ypmed.2011.10.012. 22056630 10.1016/j.ypmed.2011.10.012

[40] Grunseit AC, Chau JYY, van der Ploeg HP, Bauman A. Thinking on your feet: A qualitative evaluation of sit-stand desks in an Australian workplace. BMC Public Health. 2013;13:365. 10.1186/1471-2458-13-365. 23597291 10.1186/1471-2458-13-365 PMC3643835

[41] Prince SA, Blanchard CM, Grace SL, Reid RD. Objectively-measured sedentary time and its association with markers of cardiometabolic health and fitness among cardiac rehabilitation graduates. Eur J Prev Cardiolog. 2016;23(8):818–25. 10.1177/2047487315617101. 10.1177/2047487315617101 26607698

[42] Balducci S, D’Errico V, Haxhi J et al. Level and correlates of physical activity and sedentary behavior in patients with type 2 diabetes: A cross-sectional analysis of the Italian Diabetes and Exercise Study_2. Buchowski M, ed. *PLoS ONE*. 2017;12(3):e0173337. 10.1371/journal.pone.0173337 10.1371/journal.pone.0173337 PMC5349668 28291838

[43] English C, Healy GN, Coates A, Lewis L, Olds T, Bernhardt J. Sitting and activity time in people with stroke. Phys Ther. 2016;96(2):193–201. 10.2522/ptj.20140522. 26112254 10.2522/ptj.20140522

[44] North BJ, Sinclair DA. The intersection between aging and cardiovascular disease. Circul Res. 2012;110(8):1097–108. 10.1161/CIRCRESAHA.111.246876. 10.1161/CIRCRESAHA.111.246876 PMC3366686 22499900

[45] Pilleron S, Gower H, Janssen-Heijnen M, et al. Patterns of age disparities in colon and lung cancer survival: a systematic narrative literature review. BMJ Open. 2021;11(3):e044239. 10.1136/bmjopen-2020-044239. 33692182 10.1136/bmjopen-2020-044239 PMC7949400

[46] Sacitharan PK, Ageing. Osteoarthr Subcell Biochem. 2019;91:123–59. 10.1007/978-981-13-3681-2_6. 10.1007/978-981-13-3681-2_6 30888652

[47] Wild S, Roglic G, Green A, Sicree R, King H. Global prevalence of diabetes: estimates for the year 2000 and projections for 2030. Diabetes Care. 2004;27(5):1047–53. 10.2337/diacare.27.5.1047. 15111519 10.2337/diacare.27.5.1047

[48] Kandola A, Stubbs B, Koyanagi A. Physical Multimorbidity and sedentary behavior in older adults: findings from the Irish longitudinal study on ageing (TILDA). Maturitas. 2020;134:1–7. 10.1016/j.maturitas.2020.01.007. 32143770 10.1016/j.maturitas.2020.01.007

[49] Gunn JM, Ayton DR, Densley K, et al. The association between chronic illness, Multimorbidity and depressive symptoms in an Australian primary care cohort. Soc Psychiatry Psychiatr Epidemiol. 2012;47(2):175–84. 10.1007/s00127-010-0330-z. 21184214 10.1007/s00127-010-0330-z

[50] Franco MR, Tong A, Howard K, et al. Older people’s perspectives on participation in physical activity: a systematic review and thematic synthesis of qualitative literature. Br J Sports Med. 2015;49(19):1268–76. 10.1136/bjsports-2014-094015. 25586911 10.1136/bjsports-2014-094015

[51] Niu M, Chen J, Hou R, et al. Emerging healthy lifestyle factors and all-cause mortality among people with metabolic syndrome and metabolic syndrome-like characteristics in NHANES. J Transl Med. 2023;21:239. 10.1186/s12967-023-04062-1. 37005663 10.1186/s12967-023-04062-1 PMC10068159

[52] Yuan T, Liu H, Li XP, et al. Self-efficacy status and influencing factors among elderly patients with chronic diseases. J Jinzhou Med Univ. 2020;41(1):19–23 [in Chinese].

[53] Prochaska JJ, Spring B, Nigg CR. Multiple health behavior change research: an introduction and overview. Prev Med. 2008;46(3):181–8. 10.1016/j.ypmed.2008.02.001. 18319098 10.1016/j.ypmed.2008.02.001 PMC2288583

[54] Nagaya T, Yoshida H, Takahashi H, Kawai M. Cigarette smoking weakens exercise habits in healthy men. Nicotine Tob Res. 2007;9(10):1027–32. 10.1080/14622200701591575. 17943618 10.1080/14622200701591575

