# Supplementary material for: Self-efficacy to reduce sedentary behavior status and its influencing factors among middle-aged or older adults with chronic diseases: a cross-sectional study
Source: BMC Public Health. 2025 Dec 1;25:4201. doi: 10.1186/s12889-025-25094-w (PMC12667102; doi:10.1186/s12889-025-25094-w)
Supplement: Supplementary file 1 — Supplementary Material 1. [file 12889_2025_25094_MOESM1_ESM.docx]

**Appendix**

**Please tick "√" in the box of the question or fill in the corresponding content**

**Part 1**

**Demographic Information**

①Age: ②Gender：☐Male ☐Female

③Height(m): Weight(kg)：

⑤Education level: ☐Primary school or below ☐Junior high school

☐High school ☐College or above

⑥Work status: ☐Full time ☐Unemployed

⑦Marital status：☐ Married ☐ Single(divorced/widowed/unmarried)

⑧Per Capita Monthly Income (yuan)：☐ <3k ☐ 3-6k ☐ >6k

⑨Residence status: ☐Living alone ☐Living with children

☐Living with a spouse ☐Living with children and spouse

⑩Chronic disease status (multiple options): ☐Hypertension ☐Dyslipidemia ☐Coronary heart disease ☐Atrial fibrillation ☐Bronchiectasis ☐Asthma ☐Chronic obstructive pulmonary disease ☐Diabetes ☐Arthritis ☐Cancer ☐Not have ☐Else:

⑪Whether or smoke：☐Yes ☐No

⑫Whether lack of physical activity：☐Yes ☐No

Annotation: at least 30 minutes of physical exercise, more than 3 times per week or regular lifting, weeding, painting, picking fruits and vegetables, etc., are considered not lack of physical activity.

**Part 2**

**Self-Efficacy to Reduce Sedentary Behavior Questionnaire**

When answering the following questions, please use this definition of ***sedentary behavior***:

“Sedentary behavior is any behavior done while awake that uses a small amount of energy and occurs while sitting, reclining, or lying down.”

|  | Absolutely not confident | Not very confident | Possibly confident | Rather confident | Extremely confident |
| --- | --- | --- | --- | --- | --- |
| 1. I can stand up more often. | 1 | 2 | 3 | 4 | 5 |
| 2. I can find little ways to be less sedentary in my day. | 1 | 2 | 3 | 4 | 5 |
| 3. I can avoid being sedentary for long periods of time. | 1 | 2 | 3 | 4 | 5 |
| 4. I can be less sedentary at home. | 1 | 2 | 3 | 4 | 5 |
| 5. I can be less sedentary outside of my home (e.g., at a job or at school). | 1 | 2 | 3 | 4 | 5 |
| 6. I can be less sedentary on weekdays. | 1 | 2 | 3 | 4 | 5 |
| 7. I can be less sedentary on weekends. | 1 | 2 | 3 | 4 | 5 |
| 8. I can be less sedentary during my free time. | 1 | 2 | 3 | 4 | 5 |
| 9. I can be less sedentary when I am going to different places. | 1 | 2 | 3 | 4 | 5 |

Please rate how confident you are that you could do these things consistently if you wanted to.

**Note:** “little ways” refer to simple strategies such as talking on the phone while standing or doing housework while watching TV; “long periods of time” refers to sitting continuously for 60 minutes or more; “weekdays” generally refer to working days (typically Monday to Friday), but in contexts where official work schedules vary (e.g., due to holiday adjustments), participants were instructed to consider their actual working days; “weekends” refer to days generally free from work or study, usually Saturday and Sunday, but may vary based on individual schedules; “free time” refers to time typically used for leisure, hobbies, or relaxation.
